# Comment on unplanned out-of-hospital birth and risk factors of adverse perinatal outcome: findings from a prospective cohort

**DOI:** 10.1186/s13049-019-0626-2

**Published:** 2019-04-05

**Authors:** Razieh Bidhendi Yarandi, Mohammad Hossein Panahi

**Affiliations:** 1 Department of Epidemiology and Biostatistics, School of Public Health, Tehran University of Medical Sciences, Tehran, Iran; 2 Reproductive Endocrinology Research Center, Research Institute for Endocrine Sciences, Shahid Beheshti University of Medical Sciences, Tehran, Iran

**Keywords:** Internal validity, Methodology, Predictive factors, Prediction modelling, Sparse data bias

## Abstract

The aim of this Letter to the Editor was to report some methodological shortcomings in a recently published Article. We proved that the obtained results are subjected to the sparse data bias and presented some remedial tools such as penalization approaches. In addition, model fitting and performance aroused some controversies. In conclusion, the results of this study should be interpreted with caution and further reanalysis is necessary.

Dear Editor,

We read with great interest the recent paper by François Javaudin et.al published in the *Scandinavian Journal of Trauma, Resuscitation and Emergency Medicine* entitled “*Unplanned out-of-hospital birth and risk factors of adverse perinatal outcome: findings from a prospective cohort*” [1]. The purpose of their study was to describe neonatal morbidity and mortality, defined as death or neonatal intensive care unit hospitalization at Day 7, in a large and multicenter cohort of unplanned out-of-hospital births. Some methodological problems were mentioned in this note.

Firstly, it was not clear how predictor variables for multiple logistic regression were selected. One of the routine approaches is to select confounder variables based on a univariate model with a *p*-values less than 0.2 or 0.5 to prevent overfitting. Another approaches are directed acyclic graph, bootstrap and Bayesian model averaging.

Secondly, the authors reported multiparity as an independent predictive factor of neonatal morbidity and mortality (adjusted Odds Ratio = 70.7 [95% Confidence Interval: 4.7–1062]). A very big odds ratio with an extremely wide confidence interval proves the existence of the sparse data bias. The authors could use penalization methods such as data augmentation and Firth to resolve this problem [2–4]. Also in this study prematurity, maternal pathology and hypothermia reported as the independent predictive factors of neonatal morbidity and mortality. In fact, this conclusion is optimistic without the Internal Validity checking. Several approaches are presented for checking Internal Validity models such as split-sample validation, cross-validation and bootstrapping [5].

We suggest author provide more reliable results by the unbiased estimates and internal validity of predictors.

## References

[1] Javaudin F, Hamel V, Legrand A, Goddet S, Templier F, Potiron C, Pes P, Bagou G, Montassier E (2019). Unplanned out-of-hospital birth and risk factors of adverse perinatal outcome: findings from a prospective cohort. Scandinavian journal of trauma, resuscitation and emergency medicine.

[2] Firth D (1993). Bias reduction of maximum likelihood estimates. Biometrika..

[3] Firth D (1993). Recent developments in quasi-likelihood methods. Bulletin of the international Statistical Institute.

[4] Heinze G, Schemper M (2002). A solution to the problem of separation in logistic regression. Stat Med.

[5] Steyerberg E. Clinical prediction models: a practical approach to development, validation and updating: Springer Science & Business Media; 2008.

